# Contrast-induced acute kidney injury in patients undergoing cardiac resynchronization therapy—incidence and prognostic importance. Sub-analysis of data from randomized TRUST CRT trial

**DOI:** 10.1007/s10840-014-9887-x

**Published:** 2014-03-14

**Authors:** Jacek Kowalczyk, Radoslaw Lenarczyk, Oskar Kowalski, Tomasz Podolecki, Pawel Francuz, Patrycja Pruszkowska-Skrzep, Mariola Szulik, Michal Mazurek, Ewa Jedrzejczyk-Patej, Beata Sredniawa, Zbigniew Kalarus, for the Triple-Site Versus Standard Cardiac Resynchronization Trial (TRUST CRT) Investigators

**Affiliations:** Department of Cardiology, Congenital Heart Diseases and Electrotherapy, Silesian Center for Heart Diseases, Medical University of Silesia, ul. Curie-Sklodowskiej 9, Zabrze, Poland

**Keywords:** Cardiac resynchronization therapy, Chronic kidney disease, Contrast-induced acute kidney injury, Heart failure, Renal function

## Abstract

**Introduction:**

Because data on contrast-induced acute kidney injury (CI-AKI) in patients undergoing cardiac resynchronization therapy (CRT-D) are scarce, we aimed to assess the incidence, natural course and prognostic importance of this syndrome in CRT recipients.

**Methods:**

Study population consisted of 100 consecutive patients enrolled into the Triple Site Versus Standard Cardiac Resynchronization (TRUST CRT) trial, who were treated with CRT-D. Two patients were excluded up to 3 months after randomization and not analysed further. CI-AKI was defined as a rise in serum creatinine of at least 26.5 μmol/L (0.3 mg/dL) within 48 h after contrast exposure, or at least 50 % increase from the baseline value during index hospital stay with CRT-D implantation according to KDIGO Clinical Practice Guideline for Acute Kidney Injury.

**Results:**

Among 98 subjects of TRUST CRT trial, 10 patients (10.2 %) developed CI-AKI after CRT-D implantation. In patients with glomerular filtration rate (GFR) <60 mL/min/1.73 m^2^ on admission, the incidence of CI-AKI was almost twofold (15.4 %) higher than in subjects with GFR ≥60 (8.3 %). CRT-D recipients with CI-AKI had significantly higher mortality rate (50.0 %) compared to those without CI-AKI (17.0 %) during 30 months of follow-up (logrank *p* = 0.012). Multivariate Cox regression analysis showed CI-AKI as significant and independent risk factor for death in CRT-D recipients (hazard ratio 5.71; 95 % CI 5.16–6.26; *p* = 0.001).

**Conclusions:**

Contrast-induced acute kidney injury is a serious and frequent procedural complication of CRT-D implantation with a significant negative influence on long-term survival. The results suggest that clinical evaluation regarding renal function should be considered in CRT-D recipients, both before and after device implantation.

## Introduction

Cardiac resynchronization therapy (CRT) has become the standard of care for patients with symptomatic heart failure (HF), significant electrical dyssynchrony and moderate to severe left ventricular systolic dysfunction. Due to rapid progress in technology and broadening indications, CRT is being employed more and more widely worldwide, with the estimated implantation rate reaching 140 per million European inhabitants in 2011 [1]. According to the current recommendations, implantation of biventricular pacemaker should be preceded by detailed intraoperative assessment of coronary sinus anatomy, to allow further optimal selection of the target vessel for left ventricular lead [2]. Although alternative imaging techniques may be employed as well, balloon-occlusive contrast angiography of the coronary sinus tributaries is currently the gold standard. However, the use of contrast media, even if non-ionic and low-osmotic, is inevitably associated with the risk of contrast-induced acute kidney injury (CI-AKI)—an iatrogenic syndrome with potentially serious consequences demonstrated in some groups of patients [3–6]. Moreover, heart failure is one of the most important risk factors of CI-AKI occurrence and therefore the evaluation of this procedural complication seems to be especially important in patients receiving CRT [5–8]. In fact, data limited to the incidence of CI-AKI in a population of CRT-D recipients has been so far reported in only two studies. However, according to the authors’ best knowledge, there is a lack of data on CI-AKI prognostic value in this set of patients [8, 9]. Therefore, we aimed to assess the incidence, natural course and prognostic importance of this syndrome in CRT recipients.

## Methods

### Patients

Study population consisted of patients enrolled into the Triple Site Versus Standard Cardiac Resynchronization (TRUST CRT) trial. TRUST CRT was a single-center, single-blind, parallel, randomized, clinical trial to test the hypothesis that triple-site (double-left single-right) pacing with defibrillator is superior over conventional CRT with defibrillator [10, 11]. Eligibility criteria included heart failure in NYHA class III–IV despite optimal pharmacotherapy, left ventricular ejection fraction ≤35 %, QRS duration ≥120 ms and significant (≥40 ms) intra- or interventricular mechanical dyssynchrony. The primary end-point of the trial was the 6 months combined response rate in both groups, the secondary end-point will assess time to the first major adverse cardiac event after 3 years [10].

Between 2008 and 2010, 100 consecutive patients who met the inclusion criteria were enrolled and randomized in a 1:1 fashion to triple-site or conventional CRT with defibrillator (CRT-D). All patients were implanted with resynchronization systems with implantable defibrillator-cardioverter (InSync Sentry Model 7298, Medtronic, Minneapolis, USA). Standard, commercially available leads were used to pace/sense the atrium, right ventricle and left ventricle. A bipolar Y-connector (Lead Adaptor 2827, Medtronic) was employed to connect in parallel two left ventricular leads in the triple-site group. Atrioventricular and interventricular delays were optimized between the first and the third postoperative day under the echocardiographic guidance. Two patients were excluded up to 3 months after randomization and data of these patients were not analysed further.

The study protocol and procedural outcomes have been published previously [10, 11]. The investigation conforms with the principles outlined in the Declaration of Helsinki. The protocol of the trial was approved by the locally appointed ethics committee. Written informed consent has been obtained from all study participants.

### Procedural details

In order to analyse the potential association between postoperative renal function and characteristics of implantation procedure, data on procedural details were collected and analysed. These included procedure duration (defined as skin-to-skin time), fluoroscopy time and exposure, volume of contrast medium used and final location of left ventricular lead. Lead location was considered optimal, if its tip was in non-apical, lateral or postero-lateral segment of the left ventricle (as assessed in right- and left-lateral oblique fluoroscopic projection). Iso-osmolar, non-ionic contrast medium (Visipaque, GE Healthcare A.S., Norway) was used in all patients to perform angiography of the coronary veins. Intravenous hydration with 0.9 % sodium chloride solution (0.5 mL/kg per hour) was implemented before contrast exposure and continued for 6–12 h in all patients with glomerular filtration rate (GFR) <60. Metformin and potentially nephrotoxic drugs, e.g. NSAIDs, aminoglycosides, were avoided or discontinued for at least 48 h after contrast administration.

### Follow-up and renal function assessment

Patients were followed 1 week, 1 month, 3 months and 6 months after randomization and every 6 months thereafter. Apart from demographic, clinical data collection and echocardiographic measurements, serum creatinine levels were assessed at baseline and 6 months after randomization. In all patients, at least three blood samples were taken for serum creatinine measurements at different time points during baseline hospitalization: on admission, 1 day after implantation and at discharge. Additional, daily measurements were collected in cases of post-operative renal dysfunction, as needed. One blood sample was taken for serum creatinine 6 months after randomization. Serum creatinine levels were subsequently used to calculate GFR, according to the abbreviated Modification of Diet in Renal Disease Study Group Equation proposed by National Kidney Foundation [12]. Baseline chronic renal disease was defined as GFR <60 mL/min/1.73 m^2^ on admission. CI-AKI was defined as a rise in serum creatinine of at least 26.5 μmol/L (0.3 mg/dL) within 48 h after contrast exposure, or at least 50 % increase from the baseline value during index hospital stay with CRT-D implantation according to KDIGO Clinical Practice Guideline for Acute Kidney Injury [5, 6]. Response to CRT was defined as a reduction of at least one NYHA class after 6 months of biventricular pacing.

### Major adverse events

Data on potential adverse events were collected throughout the entire follow-up during scheduled and unscheduled visits, via telephone calls, fax and other media from patients, relatives, witnesses, death certificates, hospital records, outpatient notes, letters, device memory and all other available sources. This data was subsequently classified by two independent members of Adverse Event Adjudication Board, blinded to patients’ treatment arm. Major adverse cardiac event was considered a composite of hospitalization for heart failure, heart transplantation or all-cause death. Death certificates, hospital records and device memory data were all used to assess cardiac mortality either as sudden death or death from coronary artery disease (CAD), including myocardial infarction, congestive heart failure, valvular heart disease, cardiomyopathy, ventricular arrhythmia as well as other reasons of cardiac arrest. Remote outcome was defined as total mortality or any other endpoint occurrence within the whole 30-month observation period.

### Statistical analysis

Continuous parameters were expressed as medians with minimal and maximal values; categorical variables were presented as numbers and percentages. Comparative analysis between groups was performed using Mann–Whitney *U* test for continuous variables and Chi-square or Fisher’s exact test, as appropriate, for dichotomous parameters. Logrank tests were used to compare Kaplan–Meier curves plotted for cumulative survival. Independent predictors of death were identified with multivariate Cox regression model and expressed as hazard ratio with 95 % confidence interval. Regression models were developed after the inclusion of all parameters with univariate association with any-cause death and next backward stepwise variable selection method was performed. Multivariate logistic regression was used to identify independent predictors of CI-AKI. Generalized linear models for binomial data were constructed to test for interactions between treatment assignment (conventional vs. triple-site CRT) and prognostic effect of CI-AKI. All tests were double-sided. *p* value <0.05 was considered statistically significant. All analyses were performed using the software package Statistica (version 6.1, StatSoft Inc., Tulsa, OK, USA).

## Results

### Incidence of CI-AKI among CRT-D recipients

Among 98 subjects of TRUST CRT Trial, 10 patients (10.2 %) developed CI-AKI after CRT-D implantation. Additional analysis revealed that in patients with decreased GFR <60 on admission the incidence of CI-AKI was almost twofold (15.4 %) higher than in subjects with GFR ≥60 (8.3 %).

### Baseline and clinical characteristics of the study groups

Patients with CRT-D, who developed CI-AKI after implantation procedure, had very similar baseline and clinical characteristics when compared to those who did not develop CI-AKI (Table 1). In fact, the only significant baseline difference between these two groups was serum creatinine level on admission, which was higher in CI-AKI group. On the other hand, both groups did not differ significantly with respect to GFR on admission (Table 1). Considering data at discharge, the CI-AKI group had significantly lower GFR and higher prevalence of digoxin prescribed. All other baseline and in-hospital parameters presented in the Table 1 were very similar with non-significant differences between analysed groups. Results of multivariate logistic regression analysis showed that the only independent predictor of CI-AKI after CRT implantation was a high serum creatinine concentration at baseline (Table 2).

**Table 1:** Clinical characteristics of the study population and comparative analysis among CRT-D recipients with respect to CI-AKI occurrence

	A	B	C	*p* value (B vs. C)
	Overall population (*n* = 98)	CI-AKI (*n* = 10)	Without CI-AKI (*n* = 88)	
Baseline characteristics				
Age—years	61 (39–82)	66 (57–76)	61 (39–82)	0.13
Male gender—no. (%)	77 (78.6)	9 (90.0)	68 (77.3)	0.36
Smoking—no. (%)	20 (20.4)	2 (20.0)	18 (20.5)	0.97
Paroxysmal atrial fibrillation—no. (%)	14 (14.3)	3 (30.0)	11 (12.5)	0.14
Arterial hypertension—no. (%)	62 (63.3)	7 (70.0)	55 (62.5)	0.65
Diabetes mellitus—no. (%)	34 (34.7)	4 (40.0)	30 (34.1)	0.71
Previous myocardial infarction—no. (%)	52 (53.1)	5 (50.0)	47 (53.4)	0.84
Prior CABG—no. (%)	12 (12.2)	3 (30.0)	9 (10.2)	0.07
Previous PCI—no. (%)	37 (37.8)	4 (40.0)	33 (37.5)	0.88
Ischaemic cardiomyopathy—no. (%)	60 (61.2)	6 (60.0)	54 (61.4)	0.93
NYHA class on admission	3 (3–4)	3 (3–4)	3 (3–4)	0.37
Left ventricle ejection fraction—%	24 (12–34)	23 (17–34)	24.0 (12–34)	0.25
LVESV—mL	192 (94–481)	198 (157–321)	189 (94–481)	0.39
LVEDV—mL	260 (120–586)	272 (199–393)	255 (120–586)	0.43
Mitral EROA—mm^2^	0 (0–48)	0 (0–36)	0 (0–48)	0.93
Right ventricle diameter—mm	31 (16–42)	29 (24–42)	31 (16–42)	0.87
RVSP—mmHg	39 (11–73)	38 (13–69)	39 (11–73)	0.94
QRS complex width—ms	168 (120–220)	174 (135–206)	168 (120–220)	0.73
6 MWD—m	346 (126–488)	316 (195–414)	346 (126–488)	0.26
Peak oxygen consumption—mL/kg/min	13.1 (7.1–21.0)	13.7 (8.5–15.5)	12.9 (7.1–21.0)	0.94
NT-proBNP on admission—pg/mL	1,627 (189–28,775)	2,581 (1,077–25,890)	1,506 (189–28,775)	0.09
C-reactive protein—mg/L	2.3 (0.3–105.2)	3.1 (0.3–28.6)	2.3 (0.4–105.2)	0.96
Complete revascularization—no. (%)	68 (69.4)	7 (70.0)	61 (69.3)	0.96
Triple Site CRT—no. (%)	48 (49.0)	5 (50.0)	43 (48.9)	0.95
Renal function parameters				
Creatinine on admission—μmol/L	94 (48–285)	104 (86–180)	92 (48–285)	0.027
GFR on admission—mL/min/1.73 m^2^	72.7 (20.2–135.7)	65.6 (26.2–84.5)	73.8 (20.2–135.7)	0.08
Creatinine at discharge—μmol/L	138 (104–263)	138 (104–263)	85 (48–242)	<0.001
GFR at discharge—mL/min/1.73 m^2^	74.6 (16.9–127.7)	46.9 (16.9–67.9)	80.7 (24.4–127.7)	<0.001
Creatinine after 6 months—μmol/L	117 (71–243)	117 (71–243)	92 (52–198)	0.031
GFR after 6 months—mL/min/1.73 m^2^	73.4 (18.5–150.5)	56.8 (18.5–105.4)	75.1 (24.7–150.5)	0.059
Procedural details				
Procedure duration: skin to skin—min	110 (55–225)	115 (75–165)	110 (55–225)	0.79
Fluoroscopy time—min	20.9 (4.5–112)	19.7 (10.3–39)	20.9 (4.5–112)	0.98
Contrast media volume—mL	40 (0–155)	37 (20–125)	40 (0–155)	0.79
Use of additional technique^a^	24 (24.5)	3 (30.0)	21 (23.9)	0.67
Intraoperative decompensation^b^—no. (%)	7 (7.1)	0 (0.0)	7 (7.9)	0.35
Acute reoperation^c^—no. (%)	4 (4.1)	0 (0.0)	4 (4.5)	0.49
Optimal LV lead position—no. (%)	75 (76.5)	10 (100.0)	65 (73.9)	0.068
In-hospital-stay—days	4 (2–19)	3 (2–6)	4 (2–19)	0.11
Medication and pacing burden				
Beta-adrenergic blocker—no. (%)	97 (99.0)	10 (100)	87 (98.9)	0.74
ACE-inhibitor/ARB—no. (%)	97 (99.0)	10 (100)	87 (98.9)	0.74
Spironolactone/eplerenon—no. (%)	94 (95.9)	9 (90.0)	85 (96.6)	0.32
Loop diuretics—no. (%)	91 (92.9)	9 (90.0)	82 (93.2)	0.71
Digoxin—no. (%)	10 (10.2)	3 (30.0)	7 (8.0)	0.032
Statin—no. (%)	78 (79.6)	10 (100)	68 (77.3)	0.095
Amiodarone—no. (%)	6 (6.1)	1 (10.0)	5 (5.7)	0.59
Percentage of CRT pacing—%	99.7 (66.0–100)	99.6 (82.8–100)	99.7 (66.0–100)	0.58
Outcomes				
Responders to CRT—no. (%)	86 (87.7)	10 (100)	76 (86.4)	0.21
1-year hospitalization for HF—no. (%)	19 (19.4)	4 (40.0)	15 (17.0)	0.083
1-year MACE—no. (%)	21 (21.4)	4 (40.0)	17 (19.3)	0.13
1-year mortality—no. (%)	4 (4.1)	1 (10.0)	3 (3.4)	0.32
Remote hospitalization for HF—no. (%)	33 (33.7)	4 (40.0)	29 (33.0)	0.66
Remote MACE—no. (%)	34 (34.7)	6 (60.0)	28 (31.8)	0.079
Remote mortality—no. (%)	20 (20.4)	5 (50.0)	15 (17.0)	0.016
Cardiac mortality—no. (%)	14 (14.3)	4 (40.0)	10 (11.4)	0.016
Sudden deaths—no. (%)	4 (4.1)	2 (20.0)	2 (2.3)	0.007

*ACE* angiotensin-converting enzyme, *ARB* angiotensin receptor blocker, *CABG* coronary artery by-pass grafting, *CI-AKI* contrast-induced acute kidney injury, *CRT* cardiac resynchronization therapy, *EROA* effective regurgitant orifice area, *HF* heart failure, *GFR* glomerular filtration rate, *LV* left ventricle, *LVEDV* left ventricle end-diastolic volume, *LVESV* left ventricle end-systolic volume, *MACE* major adverse cardiac event, *6 MWD* 6-min walking distance, *NT-proBNP* N-terminal prohormone of brain natriuretic peptide, *PCI* percutaneous coronary intervention, *RVSP* right ventricle systolic pressure

Values presented as median with minimum and maximum or percentage of subjects

^a^Angioplasty or stenting within coronary vein (3 patients), use of active fixation lead (21 patients) or use of sub-selective catheters (7 patients)

^b^Requiring transient intravenous inotropic support

^c^Within the same hospital stay, due to lead dislocation (three patients) or phrenic nerve stimulation (one patient)

**Table 2:** Predictors of contrast-induced acute kidney injury after CRT implantation

Variable	Odds ratio (95 % CI)	*p* value
Procedure duration: skin to skin [1 min increase]	1.00 (0.97–1.02)	0.80
Contrast media volume [1 mL increase]	1.00 (0.97–1.03)	0.98
QRS complex width > median value [>164 ms]	1.15 (0.27–4.90)	0.85
Left ventricle ejection fraction > median value [>24 %]	0.30 (0.06–1.59)	0.15
Serum creatinine level > median value [>94 μmol/L]	6.23 (1.19–32.6)	0.03

*CI* confidence interval, *CRT* cardiac resynchronization therapy

### Natural course of CI-AKI

**Fig. 1:**
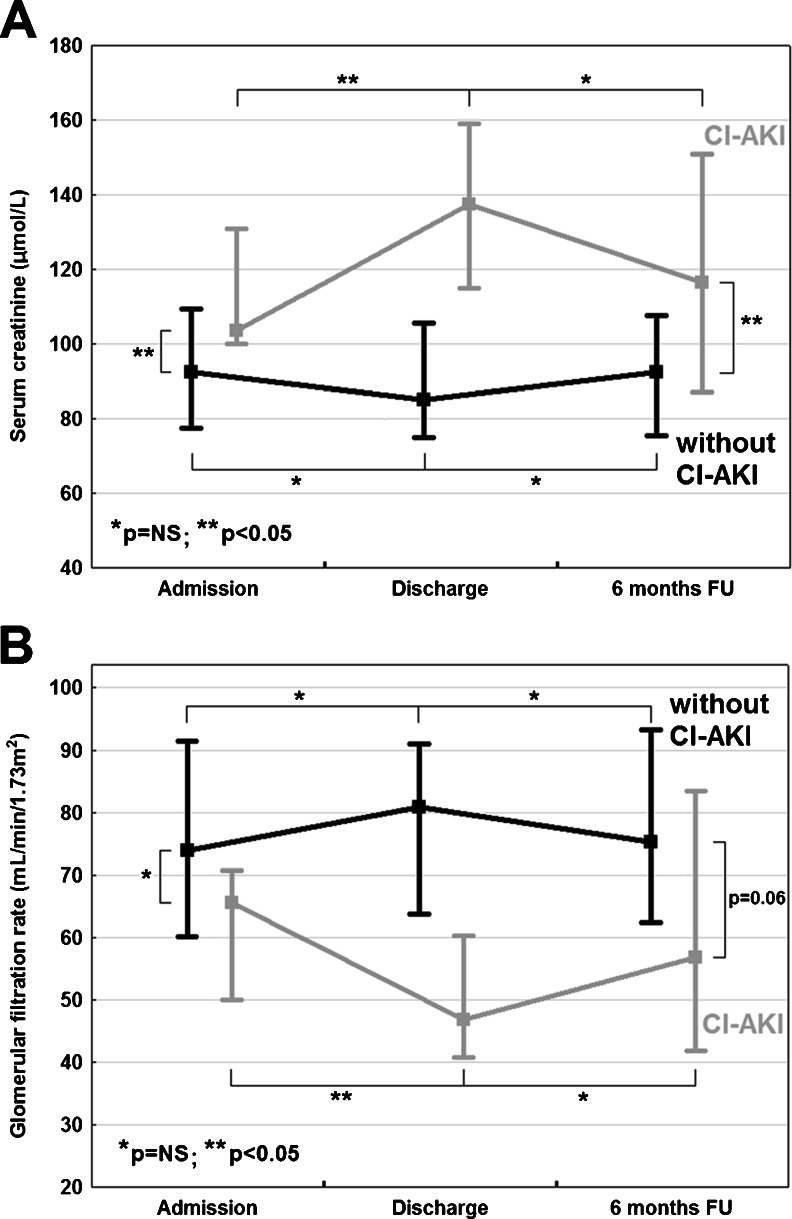
Changes in renal function estimated with serum creatinine (**a**) and glomerular filtration rate (**b**) in CRT-D recipients with respect to CI-AKI occurrence. *CI-AKI* = contrast-induced acute kidney injury; *CRT-D* = cardiac resynchronization therapy defibrillator; *FU* = follow-up; *NS* = non-significant

The analysis of natural course of renal function within particular study groups revealed that serum creatinine and median GFR did not change significantly within 6 months after CRT-D implantation (Fig. 1). The separate analysis of intergroup differences showed that, similarly to the relation at discharge, the median value of serum creatinine remained significantly higher in CI-AKI group after 6 months of follow-up. The median value of GFR after 6 months was lower in CI-AKI group with a trend towards a significant difference (Table 1).

### Prognostic value of CI-AKI

**Fig. 2:**
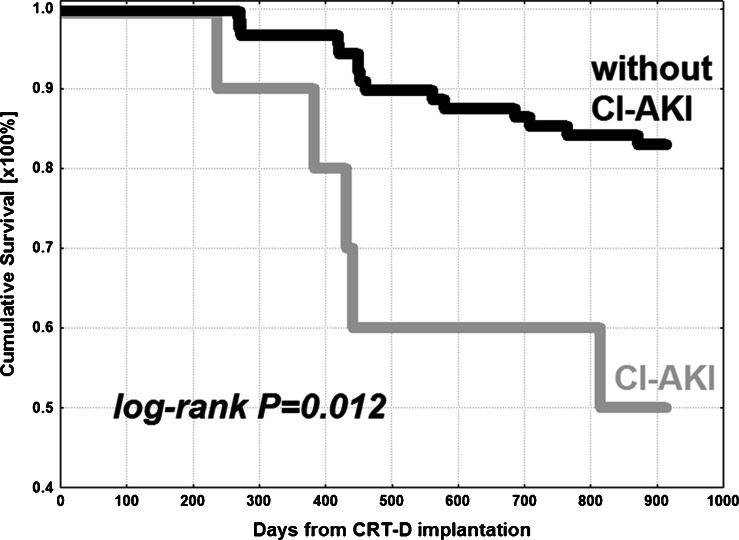
Kaplan–Meier curves for cumulative survival in CRT-D recipients with respect to CI-AKI occurrence. *CI-AKI* = contrast-induced acute kidney injury; *CRT-D* = cardiac resynchronization therapy defibrillator

CRT-D recipients with CI-AKI had significantly higher mortality rate (50.0 %) compared to those without CI-AKI (17.0 %) during 30 months of follow-up (Table 1). Cumulative surviving in analysed groups was presented with Kaplan–Meier curves and their comparison with logrank test revealed significant difference with *p* = 0.012 (Fig. 2).

All the parameters with univariate association with death and potential impact on mortality were incorporated into multivariate Cox regression model. Selection of the risk factors for all-cause death adjustment is presented in footnotes of Table 3. Multivariate Cox regression analysis using backward stepwise variable selection method showed peak oxygen consumption, QRS complex width, mitral effective regurgitant orifice area and CI-AKI as significant and independent risk factors for death in CRT-D recipients (Table 3). Prognostic effect of CI-AKI on mortality (*p* for interactions = 0.26), cardiac mortality (*p* = 0.09) and MACE (*p* = 0.92) was independent from the treatment arm (conventional vs. triple-site CRT).

**Table 3:** Independent predictors of death in CRT-D recipients

Variable	Hazard ratio (95 % CI)	*p* value
Peak oxygen consumption [1 mL/kg/min increase]	0.81 (0.71–0.91)	0.032
QRS complex width [1 ms increase]	0.97 (0.96–0.98)	0.005
Mitral EROA [1 mm^2^ increase]	1.06 (1.05–1.07)	<0.001
Contrast-induced acute kidney injury	5.71 (5.16–6.26)	0.001

Adjusted for the following: previous percutaneous coronary intervention, NYHA class on admission, NT-proBNP on admission, QRS complex width, mitral EROA, baseline peak oxygen consumption, digoxin at discharge, glomerular filtration rate at discharge, contrast-induced acute kidney injury

*CI* confidence interval, *EROA* effective regurgitant orifice area

## Discussion

The main finding of the present study is that contrast-induced acute kidney injury is a frequent complication of CRT-D implantation and has a significant negative influence on long-term survival. As it has been previously described, contrast-induced acute kidney injury, named also contrast-induced nephropathy (CIN), was a serious procedural complication with significant impact on long-term prognosis in subjects undergoing percutaneous cardiovascular procedures [5]. It has been especially well characterized in the population of patients who underwent percutaneous coronary intervention (PCI) or coronary angiography and was associated with a significant worsening of prognosis in this set of subjects [4–6, 13, 14]. Very limited data is however available on the incidence of contrast-induced acute kidney injury in the population of CRT-D recipients, and according to the authors’ best knowledge, there is no data on its prognostic value in this set of patients [8, 9].

The CI-AKI incidence of 10.2 % in our study is similar to the findings reported by other authors. Actually, there have been so far only two studies in which the frequency of CI-AKI has been assessed [8, 9]. Cowburn et al. demonstrated 14 % occurrence of contrast-induced nephropathy defined as at least 25 % increase in serum creatinine from the baseline within 48 h after contrast exposure during CRT implantation [8]. More recently, Tester et al. revealed 8 % incidence of contrast-induced nephropathy after CRT therapy implantation; however, the authors used a different definition of contrast nephropathy, which was a rise in serum creatinine of at least 25 % from the baseline ≥48 h after contrast exposure [9]. High frequency of contrast-induced nephropathy in the study of Cowburn et al. might be the result of both less common use of iso-osmolar, non-ionic contrast media and higher volumes of contrast as well as worse baseline renal function parameters [8]. For comparison, the incidence of contrast-induced acute kidney injury after PCI ranges from 2.0 % to even 20–30 % with respect to co-morbidities, and mainly depends on the baseline, pre-procedural renal dysfunction, which is one of the most important risk factors of CI-AKI development [4–7, 13, 14]. According to CIN Consensus Working Panel from 2006, contrast-induced nephropathy is responsible for approximately 11 % of hospital-acquired renal failure cases [13]. Thus, these findings coming mainly from registries, where coronary angiograms and PCI were the leading causes of CIN, are similar to the incidence of CI-AKI demonstrated in CRT recipients. Of course, the contrast volumes used during PCI are much higher than during CRT-D implantation. However, on the other hand, patients with chronic heart failure do have very often concomitant chronic kidney disease and thus are at increased risk of CI-AKI development despite relatively small amounts of contrast medium used during the procedure. Twofold increase of the incidence of CI-AKI in patients with GFR <60 on admission, demonstrated in the present study, is consistent with previous observations on increasing risk of contrast-induced nephropathy with declining baseline renal function in the population of PCI patients [15]. It has been stated that one of the most important risk factors of CI-AKI is preexisting chronic kidney disease [5, 7, 15].

Despite very similar baseline clinical characteristics of CRT-D patients, those with CI-AKI had significantly worse long-term survival than subjects without CI-AKI. Moreover, taking into account cardiac mortality, the significant difference was also observed. The aforementioned two studies on contrast-induced nephropathy after CRT presented data on the incidence of CI-AKI, however without the outcome analysis [8, 9]. Tester et al. mentioned only that the authors observed a trend towards higher long-term mortality associated with development of CIN [9]. To the authors’ best knowledge, there is currently no other data in the literature on the outcome of CRT patients with CI-AKI. It is also worth noting that after 1 year the difference in mortality, though statistically non-significant, was considerably high (10 vs. 3.4 %) with its further progression within next months. A very high 30-month mortality in CI-AKI group, almost threefold higher than in patients without CI-AKI, suggests that a very detailed clinical evaluation regarding renal function should be considered in this set of patients, both before and after CRT implantation, with implementation of all clinically proven preventive strategies against CI-AKI. Very high mortality rates in CRT-D recipients, who developed CI-AKI, might be the result of an interaction of many clinical and biological factors such as follows: predisposition to fluid overload leading to higher rates of hospitalization due to heart failure, proneness to electrolyte disturbances that may lead to arrhythmic events and sudden deaths, natural course of renal failure with its all biological consequences like abnormal vascular biology and endothelial dysfunction, hyperactivation of the renin-angiotensin system, anemia, changes in lipids, and disturbances in coagulation.

Similarly to our findings, high mortality rates in the group of CI-AKI patients were also observed by Lin et al. in CRT recipients with GFR ≤ 60 during 3 years of follow-up [16]. Some other studies have also demonstrated that renal dysfunction is an important predictor of reduced both survival and left ventricle function improvement following CRT [17–21]. The potential confirmation of this phenomenon might be sustained compromised renal function in CI-AKI group after 6 months of follow-up. Moreover, the fact that serum creatinine and median GFR did not change significantly within 6 months in particular study groups supports the hypothesis that CRT-D may have a limited impact on delaying or preventing deterioration of renal function.

We also observed that contrast exposure is a trigger for some patients with asymptomatic renal failure. Thus, CI-AKI could be a marker of patients with predisposition to overt renal failure, similarly to prediabetic states, which predispose to overt diabetes mellitus.

Another important and not reported previously finding of the present study is that CI-AKI is the strongest independent risk factor of any-cause death in CRT-D recipients, regardless of the GFR value at discharge. Other independent risk factors such as mitral regurgitation, QRS complex width and peak oxygen consumption are well-established risk predictors demonstrated in previously published studies [1, 2, 22–28]. The phenomenon of risk reduction with increasing QRS duration revealed in our study is in line with preceding reports and is explained by a high probability of substantial benefit from CRT therapy observed in CRT recipients with wider baseline QRS complex [22, 23]. The results of multivariate regression analysis with adjustment to GFR at discharge and digoxin usage—the only two differences between study groups, confirmed the finding that CI-AKI had significant impact on long-term prognosis in CRT-D recipients independent of other confounders. This observation is additionally supported by very similar baseline and clinical characteristics of both study groups.

### Study limitations

One of the potential limitations of the study is relatively small study population, which could have potentially biased the results. On the other hand, this single-centre randomized study encompassed consecutive heart failure patients, who were treated similarly both invasively (CRT-D implantation) and medically, and this fact makes the study population very homogenous and strengthens the results. The sub-analysis of data from the TRUST CRT trial, which was not primarily designed for the evaluation of CI-AKI in patients undergoing cardiac resynchronization therapy, may also bias the results. Another limitation of the study is lack of complete data from post-mortem CRT-D interrogation. Therefore, not all causes of deaths were clear, especially the sudden ones. These data would be helpful in explaining the potential reasons for different long-term prognosis and very high mortality rates in patients with CI-AKI.

In conclusion, CI-AKI is a serious and frequent procedural complication of CRT-D implantation with a significant negative influence on long-term survival independent of other risk factors. The results of the present study should be confirmed in the large cohort studies but they suggest that detailed clinical evaluation regarding renal function should be considered in CRT recipients, both before and after device implantation, with implementation of all clinically proven nephroprotective strategies.
